# Universal Sample Size Invariant Measures for Uncertainty Quantification in Density Estimation

**DOI:** 10.3390/e21111120

**Published:** 2019-11-15

**Authors:** Jenny Farmer, Zach Merino, Alexander Gray, Donald Jacobs

**Affiliations:** 1 Department of Physics and Optical Science, University of North Carolina at Charlotte, Charlotte, NC 28223, USA; jfarmer6@uncc.edu (J.F.); zmerino@uncc.edu (Z.M.); agray36@uncc.edu (A.G.); 2 Center for Biomedical Engineering and Science, University of North Carolina at Charlotte, Charlotte, NC 28223, USA

**Keywords:** density estimation, distribution free, non-parametric statistical test, decoy distributions, size invariance, scaled quantile residual, maximum entropy method, scoring function, outlier detection, overfitting detection

## Abstract

Previously, we developed a high throughput non-parametric maximum entropy method (PLOS ONE, 13(5): e0196937, 2018) that employs a log-likelihood scoring function to characterize uncertainty in trial probability density estimates through a scaled quantile residual (SQR). The SQR for the true probability density has universal sample size invariant properties equivalent to sampled uniform random data (SURD). Alternative scoring functions are considered that include the Anderson-Darling test. Scoring function effectiveness is evaluated using receiver operator characteristics to quantify efficacy in discriminating SURD from decoy-SURD, and by comparing overall performance characteristics during density estimation across a diverse test set of known probability distributions.

## 1. Introduction

**Figure 1:**
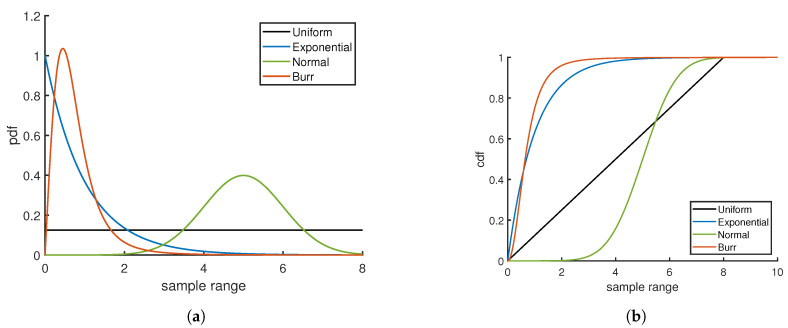
Examples of four distribution types in the form of (**a**) pdf and corresponding (**b**) cdf.

The rapid and accurate estimate of the probability density function (pdf) for a random variable is important in many different fields and areas of research [1,2,3,4,5,6]. For example, accurate high throughput pdf estimation is sought in bioinformatics screening applications and in high frequency trading to evaluate profit/loss risks. In the era of big data, data analytics and machine learning, it has never been more important to strive for automated high-quality pdf estimation. Of course, there are numerous other traditional areas of low throughput applications where pdf estimation is also of great importance, such as damage detection in engineering [7], isotope analysis in archaeology [8], econometric data analysis in economics [9], and particle discrimination in high energy physics [10]. The wide range of applications for pdf estimation exemplifies its ubiquitous importance in data analysis. However, a continuing objective regarding pdf estimation is to establish a robust distribution free method to make estimates rapidly while quantifying error in an estimate. To this end, it is necessary to develop universal measures to quantify error and uncertainties to enable comparisons across distribution classes. To illustrate the need for universality, the pdf and cumulative distribution function (cdf) for four distinctly different distributions are shown in Figure 1a,b. Comparing the four cases of pdf and cdf over the same sample range, it is apparent that the data are distributed very differently.

The process of estimating the pdf for a given sample of data is an inverse problem. Due to fluctuations in a sample of random data, many pdf estimates will be able to model the data sample well. If additional smoothness criteria are imposed, many proposed pdf estimates can be filtered out. Nevertheless, a pdf estimate will carry intrinsic uncertainty along with it. The development of a scoring function to measure uncertainty in a pdf estimate without knowing the form of the true pdf is indispensable in high throughput applications where human domain expertise cannot be applied to inspect every proposed solution for validity. Moreover, it is desirable to remove subjective bias from human (or artificial intelligence) intervention. Automation can be achieved by employing a scoring function that measures over-fitting and under-fitting quantitatively based solely on mathematical properties. The ultimate limit is set by statistical resolution, which depends on sample size.

Solving the inverse problem becomes a matter of optimizing a scoring function, which breaks down into two parts—first, developing a suitable measure that resists under- and over-fitting to the sampled data, which is the focus of this paper. Second, developing an efficient algorithm to optimize the score while adaptively constructing a non-parametric pdf. The second part will be accomplished by an algorithm involving a non-parametric maximum entropy method (NMEM) that was recently developed by JF and DJ [11] and implemented as the “PDFestimator.” Similar to a traditional parametric maximum entropy method (MEM), NMEM employs Lagrange multipliers as coefficients to orthogonal functions within a generalized Fourier series. The non-parametric aspect of the process derives from employing a data driven scoring function to select an appropriate number of orthogonal functions, as their Lagrange multipliers are optimized to accurately represent the complexity of the data sample that ultimately determines the features of the pdf. The resolution of features that can be uncovered without over-fitting naturally depends on the sample size.

Some important results in statistics [12] that are critical to obtain universality in a scoring function are summarized here. For a univariate continuous random variable, *X*, the cdf is given by $F_{X}\left(x\right)$, which is a monotonically increasing function of *x* and, irrespective of the domain, the range of $F_{X}\left(x\right)$ is on the interval (0,1). A new random variable, *R*, that spans the interval (0,1) is obtained through the mapping $r=F_{X}\left(x\right)$. The cdf for the random variable *R* can be determined as follows,

(1) $$F\left(r\right)\;=\;P\left(R\leq \;r\right)\;=\;P\left(F_{X}\left(x\right)\leq \;r\right)\;=P\left(X\leq F_{X}^{-1}\left(r\right)\right)\;=F_{X}\left(F_{X}^{-1}\left(r\right)\right)\;=r$$

Since the pdf for the random variable *R* is given as $f\left(r\right)=\frac{dF(r)}{dr}=1$ it follows that *R* has a uniform pdf on the interval (0,1). Furthermore, due to the monotonically increasing property of $F_{X}\left(x\right)$ it follows that a sort ordered set of *N* random numbers ${\left\{x_{k}\right\}}_{N}$ maps to the transformed set of random numbers ${\left\{r_{k}\right\}}_{N}$ in a 1 to 1 fashion, where *k* is a labeling index that runs from 1 to *N*. In particular, for an index $k^{\prime }>k$, it is the case that $r_{k^{\prime }}\geq r_{k}$. The 1 to 1 mapping that takes X→R has important implications for assessing the quality of a pdf estimate. The universal nature of this approach is that, for a given sample of random data and no a priori knowledge of the underlying functional form of the true pdf, an evaluation can be made of the transformed data.

Given a high-quality pdf estimate from an estimation method, ${\hat{f}}_{X}\left(x\right)$, the corresponding estimated cdf, ${\hat{F}}_{X}\left(x\right)$, will exhibit sampled uniform random data (SURD). Conversely, for a given sample from the true pdf, a poor trial estimate, ${\hat{f}}_{X}\left(x\right)$, will yield transformed random variables that deviate from SURD. The objective of this work is to consider a variety of measures that can be used as a scoring function to quantify the uncertainty in how close the estimate ${\hat{f}}_{X}\left(x\right)$ is to the true pdf based on how closely the sort order statistics of ${\hat{F}}_{X}\left(\left\{x_{k}\right\}\right)$ matches with the sort order statistics of SURD. The powerful concept of using sort order statistics to quantify the quality of density estimates [13] will be leveraged to construct universal scoring functions that are sample size invariant.

The strategy employed in the NMEM is to iteratively perturb a trial cdf and evaluate it with a scoring function. By means of a random search using adaptive perturbations, the trial cdf with the best score is tracked until the score reaches a threshold where optimization terminates. At this point, the trial cdf is within an acceptable tolerance to the true cdf and constitutes the pdf estimate. Different outcomes are possible since the method is based on a random fitness-selection process to solve an inverse problem. The role of the scoring function in the NMEM includes defining the objective target for optimizing the Lagrange multipliers, providing stopping criteria for adding orthogonal functions in the generalized Fourier series expansion and marking a point of diminishing returns where further optimizing the Lagrange multipliers results in over-fitting to the data. Simply put, the scoring function provides a means to quantify the quality of the NMEM density estimate. Optimizing the scoring function in NMEM differs from traditional MEM approaches that minimize error in estimates based on moments of the sampled data. Note that the universality of the scoring function eliminates problems with heavy tailed distributions that have divergent moments. Nevertheless, Lagrange multipliers are determined based on solving a well defined extremum problem in both cases.

**Figure 2:**
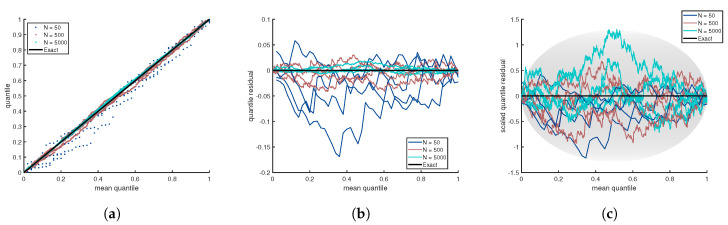
For each of the four distributions shown in Figure 1 and for sample sizes N = 50, 500, 5000 shown in all panels with same distinct colors, an empirical quantity is plotted as a function of the theoretical average quantile. The panels show (**a**) QQ-plot, (**b**) QR-plot and (**c**) SQR-plot. Only the SQR-plot is sample size invariant. As an illustration of universality in all panels, any of the colored lines could represent any one of the four distributions.

Before tackling how to evaluate the efficacy of scoring functions, a brief description is given here on how the quality of a pdf estimate can be assessed without knowing the true pdf. Visualizing a quantile-quantile plot (QQ-plot) is a common approach in determining if two random samples come from the same pdf. Given a set of *N* sort ordered random variables ${\left\{x_{k}\right\}}_{N}$ that are monotonically increasing, along with a cdf estimate, the corresponding empirical quantiles are determined by the mapping ${\left\{r_{k}\right\}}_{N}={\hat{F}}_{X}\left({\left\{x_{k}\right\}}_{N}\right)$ as described above. It is not necessary to have a second data set to compare. As described previously [11], the empirical quantile can be plotted on the y-axis versus the theoretical average quantile for the true pdf plotted on the x-axis. From single order statistics (SOS) the expectation value of $r_{k}$ is given by $\mu_{k}=k/\left(N+1\right)$ for k=1,2,...N, which gives the mean quantile. Figure 2a illustrates the QQ plot for the distributions shown in Figure 1. The benefit of the QQ plot is that it is a universal measure. Unfortunately, for large sample sizes, the plot is no longer informative because all curves approach a perfect straight line as random fluctuations decrease with increasing sample size. A quantile residual (QR) allows deviations from the mean quantile to be readily visualized when one sample size is considered. However, as illustrated in Figure 2b, the residuals in a QR-plot decrease as sample size increases. Hence, the quantile residual is not sample size invariant.

The QR-plot is scaled [11] in such a way as to make the scaled quantile residual (SQR) sample size invariant. From SOS, the standard deviation for the empirical quantile to deviate from the mean quantile is well-known to be $\sigma_{k}=\sqrt{\mu_{k}\left(1-\mu_{k}\right)}/\sqrt{N+2}$ where *k* is the sort order index. Interestingly, all fluctuations regardless of the value for the mean quantile scale with sample size as $1/\sqrt{N+2}$. Sample size invariance is achieved by defining SQR as $\sqrt{N+2}\left(r_{k}-\mu_{k}\right)$ and, when plotted against $\mu_{k}$, one obtains a SQR-plot. Figure 2c shows an SQR-plot for three different sample sizes for each of the four distributions considered in Figure 1. It is convenient to define contour lines using the formula $sf\sqrt{\mu (1-\mu )}$, where the scale factor, sf, can be adjusted to control how frequently points on the SQR plot will fall within a given contour. In particular, 99% of the time the SQR points will fall within the boundaries of the oval when bounded by $\pm 2.58\sqrt{\mu (1-\mu )}$. Scale factors of 1.65, 1.96, 2.58 and 3.40 lead to 90%, 95%, 99% and 99.9% of SQR points falling within the oval based on numerical simulation. Interestingly, the scale factors of 1.65, 1.96, 2.58 and 3.40 respectively correspond to the *z*-values of a Gaussian distribution at the 90%, 95%, 99% and 99.9% confidence levels.

The SQR-plot provides a distribution free visualization tool to assess the quality of a cdf estimate in three ways. First, when the SQR falls appreciably within the oval that encloses 99% of the residual, it is not possible to reject the null hypothesis. Second, when the SQR exhibits non-random patterns, this is an indication of systematic error introduced by the estimator method. Finally, when the SQR has suppressed random fluctuations such that it is close to 0 for an extended interval, this indicates that the pdf estimate is over-fitting to the sample data. In general, over-fitting is hard to quantify [14]. As the graphical abstract shows, it is possible to plot the SQR against the original random variable *x* instead of the mean quantile. Doing this deforms the oval or "lemon drop" shape of the SQR-plot but it directly shows where problems in the estimate are locally occurring in relation to the pdf estimate. The aim of this paper is to quantify these salient features of an SQR-plot using a scoring function.

This work was motivated by the concern that different scoring functions will likely perform differently in terms of speed and accuracy in NMEM. The scoring function that was initially considered was constructed from the natural logarithm of the product of probabilities for each transformed random variable, given by ${\hat{F}}_{X}\left(\left\{x_{k}\right\}\right)$. This log-likelihood scoring function provides one way to measure the quality of a proposed cdf. Interestingly, the log-likelihood scoring function has a mathematical structure similar to the commonly employed Anderson-Darling (AD) test [15,16]. As such, the current study considers several alternative scoring functions that use SQR and compares how sensitive they are in quantifying the quality of a pdf estimate. Other types of information measures that use cumulative relative entropy [17] or residual cumulative Kullback–Leibler information [18,19] are possible. However, these alternatives are outside the scope of this study, which focuses on leveraging SQR properties. The scoring function must exhibit distribution free and sample size invariant properties so that it can be applied to any sample of random data of a continuous variable and also to sub-partitions of the data when employed in the PDFestimator. It is worth noting that all the scoring functions presented in this paper exhibit desirable properties with similar or greater efficacy than the AD scoring function and all are useful for assessing the quality of density estimates.

In the remainder of this paper, a numerical study is presented to explore different types of measures for SQR quality. The initial emphasis is on constructing sensitive quality measures that are universal and sample size invariant. These scoring functions based on SQR properties can be applied to quantifying the accuracy (or ‘’goodness of fit”) of a pdf estimate created by any methodology, without knowledge of the true pdf. The SQR is readily calculated from the cdf which is obtained by integrating the pdf. To determine which scoring function best distinguishes between good and poor cdf estimates, the concept of decoy SURD is introduced. Once decoys are generated, Receiver Operator Characteristics (ROC) are employed to identify the most discriminating scoring function [17]. In addition to ROC evaluation, performance of the PDFestimator for different plugged in scoring functions is evaluated. This benchmark is important because the scoring function is expected to affect the rate of convergence toward a satisfactory pdf estimate using the NMEM approach. After discussing the significance of the results, several conclusions are drawn from an extensive body of experiments.

## 2. Results

### 2.1. Sample Size Invariant Scoring Functions

Seven scoring functions are defined in Table 1. At the moment, the input to these scoring functions is SURD of sample size *N*. Specifically, *N* random numbers are independently and identically drawn uniformly on the interval (0, 1) and then sort ordered to give SOS represented by the set ${\left\{r_{k}\right\}}_{N}$ where $0<r_{k}\leq r_{k+1}<1\;\forall \;k=1,2,...N$. For sample size, *N*, a scoring function of type *t* is evaluated as $S_{t}\left({\left\{r_{k}\right\}}_{N}\right)$, which defines a new random variable that is simply denoted as $S_{t}\left(N\right)$. A scoring function is scale invariant if the probability density for $S_{t}\left(N\right)$ is independent of sample size, which typically holds only for large *N*. However, finite size corrections are made for each scoring function and are listed in Table 1. In all cases, the finite size corrections are empirically determined based on numerical simulation to achieve approximate scale invariance for N≥9. In all coefficients reported, there is a (3) error in the last significant figure, such as 0.406(3) or 11.32(3).

As defined in Table 1, the proposed scoring functions include the relevant part of the Anderson-Darling (AD) measure [15], denoted as $S_{AD}$, and the quasi log-likelihood formula [11], denoted as $S_{LL}$. Note that $S_{LL}=\;log\left[\prod_{k}p_{k}{(r}_{k})\right]$ where $p_{k}\left(r_{k}\right)$ is the exact pdf corresponding to a beta distribution that describes the random variable $r_{k}$ as derived from SOS [13]. The quasi log-likelihood is not an exact log-likelihood. Rather, $S_{LL}$ corresponds to a mean field approximation where correlations between the random variables, ${\left\{r_{k}\right\}}_{N}$, are neglected. Another scoring function is defined as $S_{VAR}=\langle z_{k}^{2}\rangle$, where $z_{k}=\left(r_{k}-\;\mu_{k}\right)/\sigma_{k}$. As mentioned in the Introduction, $\mu_{k}=\langle r_{k}\rangle =k/\left(N+1\right)$ is the mean quantile of the k-th random variable and $\sigma_{k}=\;\mu_{k}\left(\mu_{k}-1\right)/\sqrt{N+2}$ is the standard deviation of the k-th random variable about its mean. Essentially $S_{VAR}$ is the mean variance of a “z-value” for SOS.

**Figure 3:**
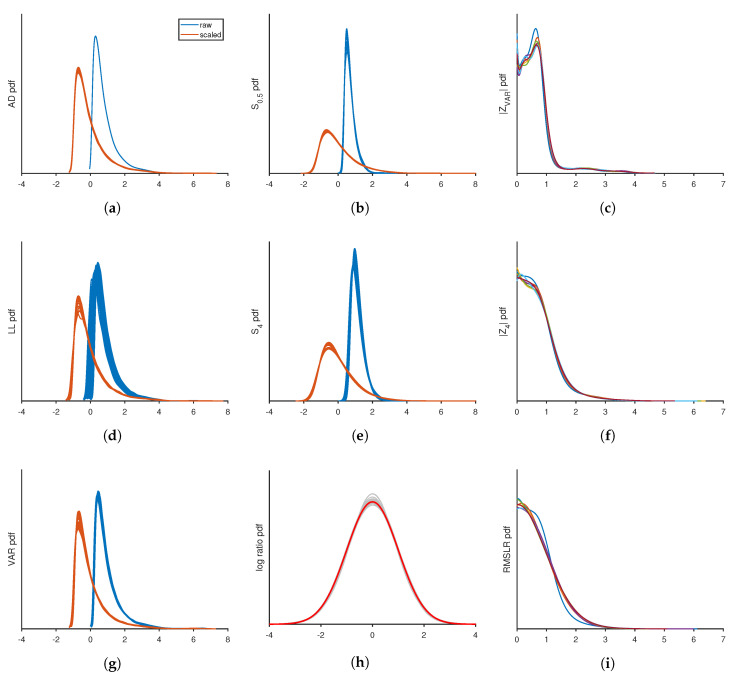
Illustration of sample size invariance in the probability density function for various scoring functions. The sample sizes selected in panels (**a**,**b**,**d**,**e**,**g**,**h**) to show data collapse include N = 9, 11, 12, 14, 17, 20, 24, 33, 49, 95, 110, 124, 142, 166, 199, 249, 332, 497, 990, 1500, 2015, 3298, 5505, 8838, 14,467, 23,684, 38,771, 63,471, 103,905, 272,389, 750,000, 1,000,000, 2,000,000. The sample sizes selected in panels (**c**,**f**,**i**) include N = 10, 50, 200, 1000, 5000, 20,000, 100,000. In panel h, the results from each system size along with all partitions made within each system size (a total of 111 cases) is plotted as light gray lines. The red line shows the result of a normal distribution, indicating that the scaling is well described by a normal distribution. All other details are described in the text.

Despite sharing a similar mathematical form, the $S_{AD}$ and $S_{LL}$ scoring functions are not the same, even in the limit N→∞. At face value, these functions look very different. However, after shifting the origin of these functions to their natural reference points and scaling $S_{LL}$ by a factor of −2, which was empirically determined to obtain data collapse, these two measures were remarkably similar. To demonstrate this, let $S_{AD}^{\prime }\equiv \;S_{AD}\left(\left\{r_{k}\right\}\right)\;-\;S_{AD}\left(\left\{\mu_{k}\right\}\right)$ and $S_{LL}^{\prime }\equiv \;-2\left[S_{LL}\left(\left\{r_{k}\right\}\right)\;-\;S_{LL}\left(\left\{\mu_{k}\right\}\right)\right]$. The natural reference points $S_{AD}^{o}$ and $S_{LL}^{o}$ are respectively defined as $S_{AD}$ and $S_{LL}$, evaluated at the mean quantiles. Figure 3a,d show the pdf for $S_{AD}^{\prime }$ and the pdf for $S_{LL}^{\prime }$ are approximately sample size invariant and markedly similar. Interestingly, $S_{AD}^{\prime }$ has superior sample size invariance because it reaches its asymptotic limit extremely fast, as reported almost 60 years ago [20].

To improve or create a scale invariant scoring function, finite size corrections are incorporated by transforming $S_{t}\left(N\right)$ to a z-value. For all score types, $Z_{t}=\;\left(S_{t}-\mu_{t}\right)/\sigma_{t}$ where $\mu_{t}$ is the average of $S_{t}$ and $\sigma_{t}$ is the standard deviation of $S_{t}$ about its mean. All shifts and scale factors used to transform $S_{t}\left(N\right)\to Z_{t}\left(N\right)$ are given in Table 1. Figure 3a,d,g show that, after finite size corrections, the pdf for the three scoring functions $Z_{AD}$, $Z_{LL}$ and $Z_{VAR}$ exhibit excellent scale invariance. Furthermore, the pdf for these scoring functions fall on top of one another in a massive data collapse (data not shown) indicating they share the same pdf for all practical purposes. It is worth noting that because this is a numerical study, there is uncertainty in the formulas that define the corrections to finite sample size. As can be clearly seen in Figure 3a, the AD measures before finite size corrections are applied display the most impressive data collapse. Indeed, the observed data collapse from numerical simulation are tighter than the intrinsic uncertainties in the correction to finite size samples. In contrast, the log likelihood measure has the most dispersion in its data collapse before finite size corrections are applied. In this case, the finite sample size corrections greatly improved the data collapse.

The most surprising result is that this numerical study demonstrates that $Z_{VAR}$ shares the same pdf as $Z_{AD}$. This result is surprising because both $Z_{AD}$ and $Z_{LL}$ involve linear combinations of logarithms, while $Z_{VAR}$ has no logarithms. However, it is not surprising that $Z_{VAR}$ has good scaling properties because the function is defined in terms of the scaled variable, otherwise called the z-value. The transformation to the z-value naively sets the mean to the origin and normalizes the variance. As such, it would be somewhat surprising if $Z_{VAR}$ did not exhibit data collapse as a function of the z-value. Given that $Z_{VAR}$ scales, it is expected that generalized moments of the z-value variable will exhibit data collapse and also exhibit sample size invariance.

From a practical standpoint, it is computationally faster to work with $Z_{VAR}$. Therefore, additional scoring functions defined as $S_{p}={\langle |z_{k}{|}^{p}\rangle }^{1/p}$ for $p=\frac{1}{2}\;,\;1,\;2,\;3,\;4$ were considered. Note that $S_{2}$ is the standard deviation of $z_{k}$ and, after finite size corrections are applied, $S_{p}\to Z_{p}$. The cases $p=\frac{1}{2}$ and p=4 are listed in Table 1 and exhibit scale invariance as shown in Figure 3b,e respectively. The p=1,2,3 cases (data not shown) are similar and straddle the limiting cases smoothly. It is worth mentioning that the natural reference at the mean quantile is zero for $S_{VAR}$ and $S_{p}$.

By exploring SURD for additional patterns, it was observed that two disjoint blocks of the same size can be compared using double order statistics (DOS). Among all random variables, ${\left\{r_{k}\right\}}_{N}$, the indices that span from $k_{o}^{1}$ to $k_{f}^{1}$ define block 1 and the indices that span from $k_{o}^{2}$ to $k_{f}^{2}$ define block 2. Without loss of generality, block 2 is taken to be to the right of block 1, such that $k_{o}^{1}<k_{f}^{1}<\;k_{o}^{2}<k_{f}^{2}$. With *m* random variables in both blocks, m−1 differences given by $\delta_{k}=r_{k+1}-\;r_{k}$ are used in the scoring function $S_{LR}^{2,1}=\langle log\left(\delta_{k}^{2}/\delta_{k}^{1}\right)\rangle$, which simplifies to $S_{LR}^{2,1}=\;\langle log\left(\delta_{k}^{2}\right)\rangle -\langle log\left(\delta_{k}^{1}\right)\rangle$. Importantly, $\langle log\left(\delta_{k}^{j}\right)\rangle$ is calculated for all disjoint blocks at once. By partitioning all random variables into equal blocks of indices, the mean log-ratio is calculated rapidly for all pairs of blocks. For any size block and for any pair of blocks, $S_{LR}^{\left(i,j\right)}$ exhibits strong scale invariance as shown in Figure 3h. Over a hundred diverse cases are shown as gray lines. Interestingly, the pdf for $S_{LR}^{\left(i,j\right)}$ is essentially a normal distribution shown as a red line.

Because $S_{LR}^{\left(i,j\right)}$ is localized to a pair of blocks, to cover the entire SQR-plot a new scoring function is constructed by taking the root mean square of all distinct pairs of $S_{LR}^{\left(i,j\right)}$. For a calculation time proportional to sample size, the size of a block is set proportional to $\sqrt{N}$, which necessarily makes the number of blocks, $N_{b}$, proportional to $\sqrt{N}$. The pdf for RMSLR is nearly sample size invariant as shown in Figure 3i. From Table 1, it appears the finite size corrections for RMSLR are complicated. However, as will be discussed below, scale invariance should be preserved for sub-samples of the data, called partitions. It turns out that only RMSLR requires special attention to make partitions scale, where $N_{p}$ is the number of data points being sub-sampled. Finally, the absolute value of the measures $Z_{VAR}$ and $Z_{4}$ are respectively shown in Figure 3c,f. Note that taking an absolute value of a measure that is scale invariant will remain scale invariant.

### 2.2. Redundant and Complimentary Information

**Figure 4:**
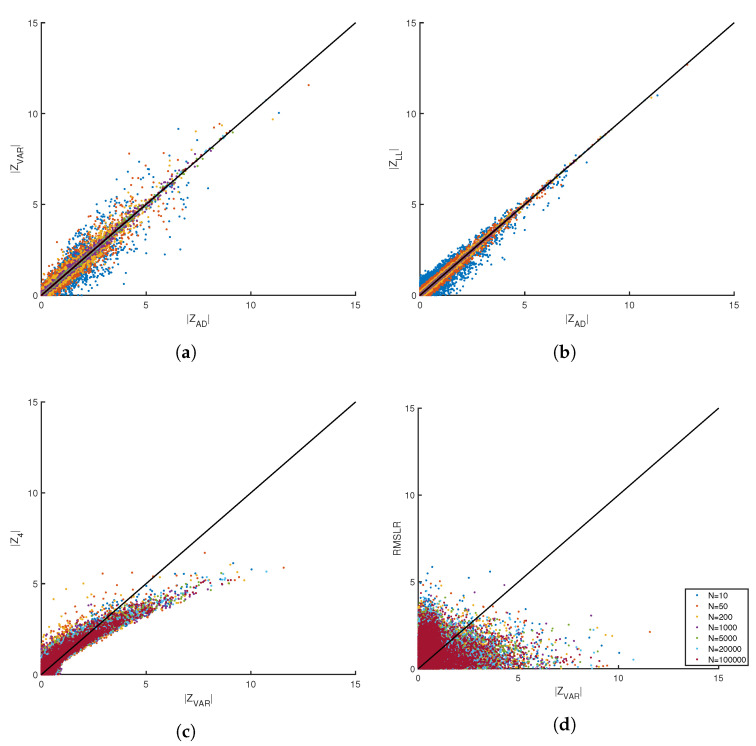
Examples of pairwise comparisons of different measures through scatter plots. (**a**,**b**) show that the |*Z_AD_*| measure is statistically the same as the |*Z_LL_*| and |*Z_VAR_*| measures. (**c**) Shows mild differences between |*Z*_4_| and |*Z_VAR_*|. (**d**) Shows that the information content between RMSLR and |*Z_VAR_*| is very different.

Since the pdf of different scoring functions may be similar or the same, the next question addressed is how do different measures compare when applied to the same SURD? For sample size *N*, SURD is generated using numerical simulation and each measure is evaluated per realization of ${\left\{r_{k}\right\}}_{N}$. For 100,000 random trials per *N*, a 1 to 1 comparison is made between $Z_{a}\left(N\right)$ versus $Z_{b}\left(N\right)$ with a≠b. Note that by definition, $Z_{t}\left(N\right)$ has a mean of zero and a standard deviation of 1. For reasons that will become clear below, absolute values are taken on the scoring functions. Despite the pdf for $|Z_{VAR}|$, $|Z_{LL}|$ and $|Z_{AD}|$ being practically identical for all sample sizes, scatter plots indicate that the scores are not identical on a 1 to 1 basis. Figure 4a,b plot $|Z_{VAR}|$ and $|Z_{LL}|$ against $|Z_{AD}|$, respectively. Although there is always a tight linear correlation, there is more scatter in the comparison at smaller sample sizes. As N→∞ the different scores converge to the same value, although the approach to the asymptotic limit for each measure differs. These differences have important implications for application to density estimation as discussed below.

The scatter plot of $\left|Z_{4}\right|$ versus $|Z_{VAR}|$ in Figure 4c shows that these two measures characterize SURD in a fundamentally different way due to the strong deviation of $\left|Z_{4}\right|$ relative to $|Z_{VAR}|$ with modest statistical scatter. The greatest non-linear deviation between the two scores occurs at large values of $|Z_{VAR}|$, corresponding to outliers in SURD. The scatter plot of RMSLR versus $|Z_{VAR}|$ in Figure 4d shows strong random scatter with no discernible deterministic dependence. Hence, RMSLR and $|Z_{VAR}|$ measure different SURD characteristics. Yet, despite their conspicuous differences, the pdf for $\left|Z_{4}\right|$ and RMSLR are qualitatively similar as shown in Figure 3f,i, respectively.

As demonstrated by scatter plots, various scoring functions characterize SURD in different or similar ways relative to one another. Note that combining measures with complimentary properties can potentially lead to a more sensitive measure. Through reductive analysis, a composite score (CS) is proposed as:

(2) $$CS=|Z_{VAR}+0.666|+\left[\max\left(2.5,|Z_{4}|,RMSLR\right)-2.5\right]$$

In constructing CS, the most probable score for $Z_{VAR}$, near 0.666, is used as a baseline. Then contributions are added from outliers from either $|Z_{4}|$ or RMSLR, whichever is larger. The last term does not modify the score when no outlier is detected, otherwise the contribution to CS continuously increases starting at zero at just above the threshold for outlier detection.

### 2.3. Partition Size Invariance

**Figure 5:**
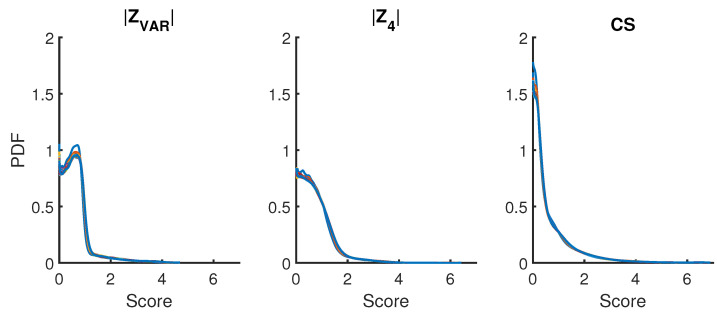
$Z_{VAR}$, $|Z_{4}|$ and CS illustrate the three most distinct measures considered. Data collapse based on the probability density for different measures is demonstrated for N= 10, 50, 200, 1000, 5000, 20,000, 100,000 in addition to $N_{p}=$ 1025, 2049, 4097, 8193, 16,385, 32,769, 65,537. A different color is used for each sample size.

A critical part of the algorithm in the PDFestimator [11] is that the input data sample is partitioned into hierarchical sub-samples by powers of 2 when N>1025. Consequently, the employed scoring function should be sample size invariant for all partitions. Invariance of partition size, $N_{p}$, is satisfied by all scoring functions described in this work, as exemplified in Figure 5 for three of the most distinct measures. Furthermore, for any realization of SURD of size *N*, all partitions within have essentially the same score independent of the type of scoring function.

A necessary requirement for all the scoring functions is that sub-sampling must be uniformly distributed over the data. It is worth noting that $S_{AD}$ (and its corresponding $Z_{AD}$) is particularly sensitive to the way the uniform sub-sampling is performed within a partition. Due to the form of the $S_{AD}$ equation, it is critical that the selected points are symmetric about the center index in the sort ordering. The number of samples used in a partition is always odd of the form $N_{p}=1+2^{n}$. Thus, the median point is included and for each index selected to be in the sub-sample below the median, a corresponding mirror image index above the median is selected. For example, if there are 17 indices in the full sample, indices 1, 4, 9, 14, 17 has the required mirror symmetry. All other scoring functions are not sensitive to breaking mirror symmetry.

### 2.4. Decoy SURD

For the purpose of quantifying how well a scoring function discriminates between true SURD and random data that is not SURD, a controlled decoy-SURD (dSURD) is generated. Let $\left\{r_{k}^{o}\right\}$ define SURD and let $\left\{r_{k}^{d}\right\}$ define dSURD. As described in detail in Section 4.4, a decoy cdf, $F_{d}\left(r\right)$, is constructed to facilitate the 1 to 1 mapping given by $\left\{r_{k}^{d}\right\}=F_{d}\left(\left\{r_{k}^{o}\right\}\right)$. If $F_{d}\left(r\right)=r$, then the output is identical to the input. A decoy-SURD is controlled by adding a perturbation of the form $F_{d}\left(r\right)=r+\Delta \left(r\right)$. By choosing various functional forms for the perturbation and, by controlling the amplitude of the perturbation, it is a simple matter to make a broad spectrum of decoys that range from impossible to markedly obvious to detect at any specified sample size.

**Figure 6:**
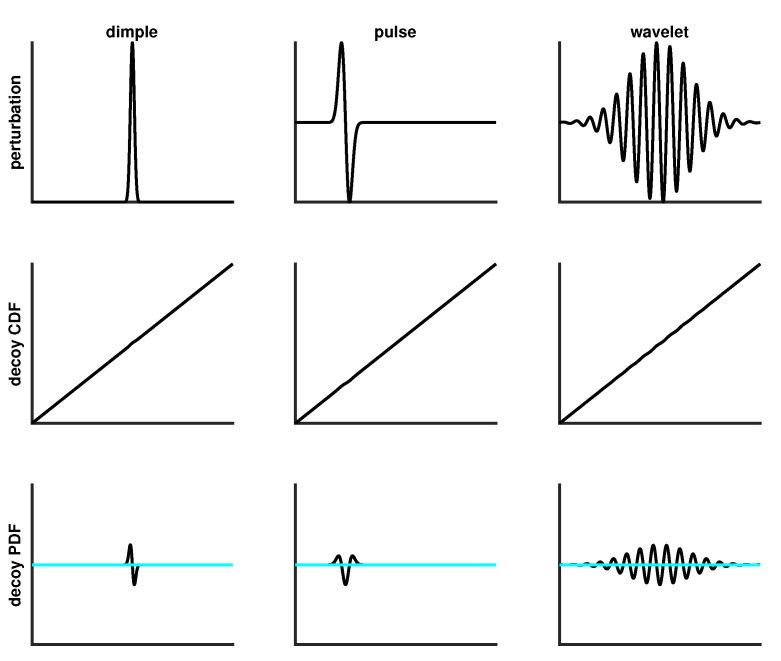
Top row shows three examples of localized perturbations for moderately difficult decoys. Center row shows the corresponding cdf. Bottom row shows the pdf, where the cyan horizontal highlights the probability density function (pdf) for sampled uniform random data (SURD).

**Figure 7:**
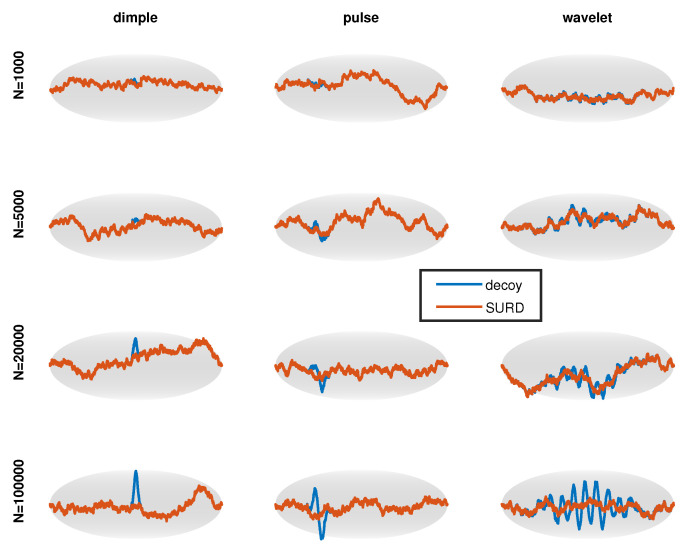
Progression of scaled quantile residual (SQR)-plots for moderately difficult localized decoys as sample size increases.

In Figure 6, the middle row shows the decoy cdf resulting from the perturbations shown along the top row. This is an example of a moderately hard dSURD because by eye the decoy cdf looks close to a perfect straight line. To make it clear that dSURD is indeed different from SURD, the pdf for each case is shown along the bottom row. For a sufficiently large sample size, statistical resolution will be good enough to resolve these small perturbations, but for smaller sample sizes the perturbation will not be detectable. To demonstrate how statistical resolution increases with larger sample sizes, Figure 7 shows SQR-plots for SURD and its corresponding dSURD for samples sizes of 1000, 5000, 20,000 and 100,000. These three cases are examples of localized perturbations.

**Figure 8:**
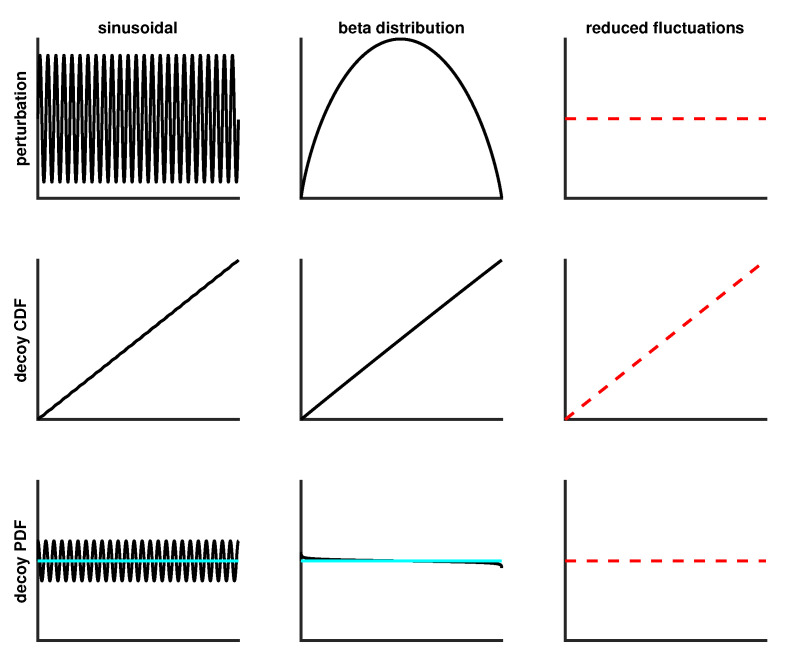
Extended perturbations for moderately difficult decoys. The cyan horizontal line shown on the bottom panels defines the pdf for SURD. The red dashed lines represent suppression of fluctuations.

**Figure 9:**
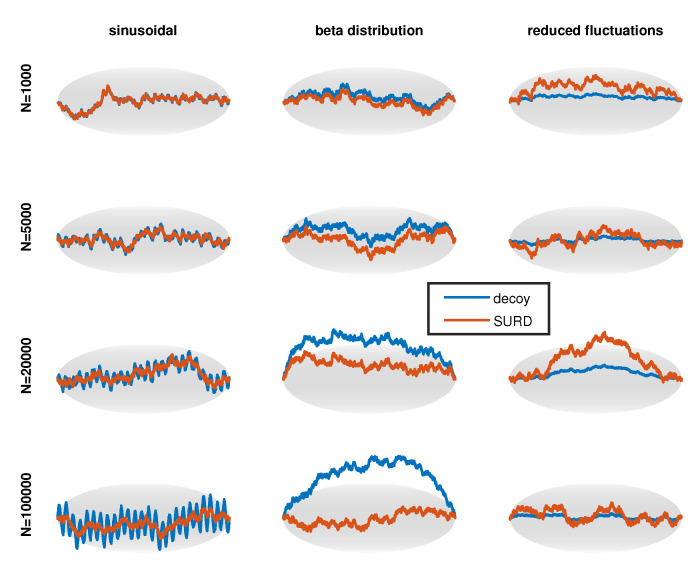
Progression of SQR-plots for moderately difficult extended decoys as sample size increases.

Three additional perturbations of an extended type are shown in Figure 8 using the same layout. The last column plots the perturbation, cdf and pdf as dashed red lines because “reduced fluctuation” is a special type of perturbation that is also explained in Section 4.4. As the name implies, fluctuations are suppressed, representing a scenario where a pdf estimate over-fits the data. Figure 9 shows the SQR-plots for SURD and its corresponding dSURD of the extended type for samples sizes of 1000, 5000, 20,000 and 100,000. Note that the reduced fluctuation perturbation is equally detectable at any size sample because fluctuations are suppressed by a fixed proportion in relation to true SURD.

By comparing measures applied to dSURD and SURD, it can be expected that the more sensitive scoring function is one that detects a given perturbation at smaller sample sizes compared to other scoring functions. It is also expected that a certain scoring function will be able to detect certain types of perturbations more readily than other types of perturbations. As such, it is likely impossible to find a perfect scoring function that performs best on all decoy types all the time. Nevertheless, for a given diverse set of dSURD examples, the best overall performing scoring functions with the greatest sensitivity or selectivity can be deduced using receiver operator characteristics.

### 2.5. Receiver Operator Characteristics

**Figure 10:**
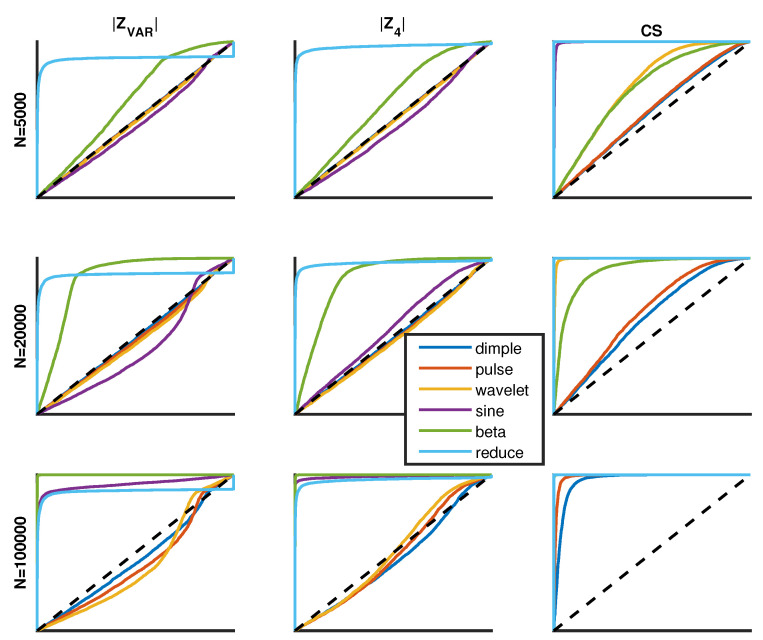
The qualitative features of receiver operator characteristic (ROC) curves are shown for sample sizes of 5000, 20,000 and 100,000 along the top, middle and bottom rows. The left, middle and right columns correspond to the $|Z_{VAR}|$, $|Z_{4}|$ and CS scoring functions. The (y-axis, x-axis) corresponds to the fraction of true (positives, negatives) having a range from 0 to 1. Each ROC curve compares 6 different decoy types.

Receiver operator characteristics (ROC) are calculated based on simulation data involving 10,000 trials of SURD over a broad range of *N* samples, and for each SURD, many dSURD mappings are generated for each of the six decoy types shown above. Results are exemplified in Figure 10, showing ROC curves for three different sample sizes and six different decoy types. ROC curves quantify the efficacy of a scoring function in discriminating SURD from dSURD. Figure 10 shows representative results for moderately difficult decoys. As a point of reference, easy, moderate and hard decoys are aimed at requiring about 1000, 10,000 and 100,000 samples to have sufficient statistical resolution to notice dSURD just barely by eye (e.g., see Figure 7 and Figure 9). Only the decoy that reduces fluctuations using a fixed scale factor has the same difficulty for detection independent of sample size.

It is common practice to quantify ROC curves by their area under the curve (AUC). Table 2 gives all AUC values for all the cases shown in Figure 10. The ROC curves and the results listed in Table 2 clearly show that CS detects decoys better than the other measures. Of course, informed by reductive analysis, this result was purposely intended during the construction of CS given in Equation (2). In summary, it is generally found that $Z_{AD}$, $Z_{LL}$, $Z_{VAR}$, $|Z_{AD}|$, $|Z_{LL}|$, $|Z_{VAR}|$, $Z_{4}$, $|Z_{4}|$ and CS scoring functions are all good measures to distinguish SURD from easy to detect dSURD. However, it is always possible to create decoy SURD that will go undetected by any measure (e.g., Figure 10).

In general, $Z_{AD}$, $Z_{LL}$ and $Z_{VAR}$ share similar ROC curves and $|Z_{VAR}|$ and $|Z_{4}|$ have similar ROC curves. The most sensitive scoring function is CS. The reason $Z_{t}$ and $|Z_{t}|$ are considered as two separate cases is now easily explained. First note that $Z_{t}$ has a mean of zero and a standard deviation of 1. For a decoy type of "reduced fluctuations" that mimics an over-fitting scenario, the ROC curve becomes inverted for any type of measure, $Z_{t}$. However, the inversion problem is eliminated when considering $|Z_{t}|$ because both over-fitting and under-fitting is detected when $|Z_{t}|$ is large. Finally, only the combined score, CS, readily detects very localized perturbations due to its RMSLR component.

### 2.6. PDF Estimation Performance

**Figure 11:**
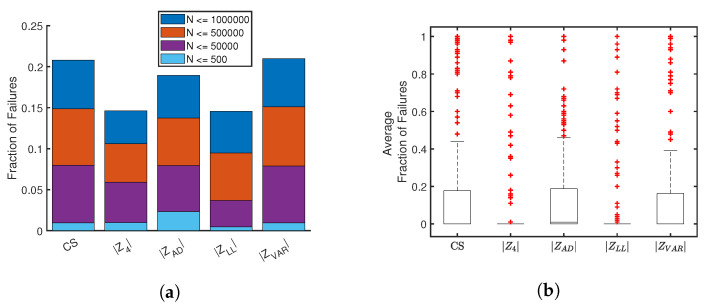
Figure (**a**) cumulative averages of failure rates across four ranges Figure (**b**) distribution of failure rates for each scoring method. Box plots show inner-quartiles and whiskers represent range of data excluding outliers, which are shown as red crosses.

Figure 11 summarizes the comparative statistics for failure rates. The bar plots in Figure 11a report averages across distributions and random samples, for cumulative ranges of sample sizes. As expected, the failure rate increases with sample size. For all scoring methods, average failure rates are typically on the order of 10% for sample sizes less than one million. Failure rate averages are the least for $|Z_{4}|$ and $|Z_{LL}|$, a trend that holds across sample size. The associated box plots in Figure 11b more clearly demonstrate the computational advantage of $|Z_{4}|$ and $|Z_{LL}|$ over the other scoring methods. All scoring methods have between 50 and 60 outliers, but $|Z_{4}|$ and $|Z_{LL}|$ have virtually no failures outside of these extreme values.

**Figure 12:**
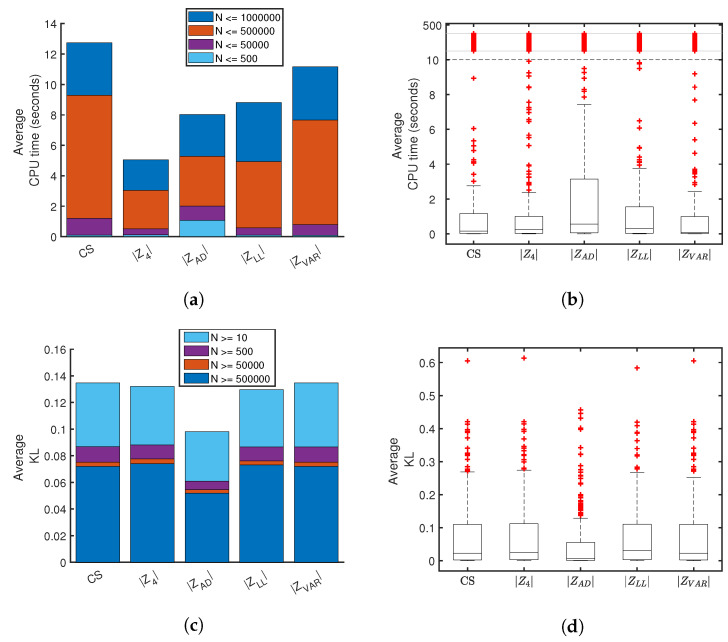
Comparative statistics between five scoring methods averaged over successful solutions. Cumulative averages for (**a**) performance time across four sample size ranges and (**c**) Kullback-Leibler divergence [21]. Panels (**b**,**d**) show box plots for the respective data shown in panels (**a**,**c**). Box plots show inner-quartiles and whiskers represent range of data excluding outliers, which are shown as red crosses.

For computational time and Kullback-Leibler (KL) divergence [21], or simply KL, care must be taken to ensure a fair comparison, accounting for failure rates. Thus, a subset of the data is considered for these measurements. Of the 275 test sets (25 distributions at 11 sample sizes), 230 of these contain at least 10 successes out of the 100 trials, across all five scoring methods. The remaining 45 tests contain failure rates greater than 90% for at least one scoring method and are eliminated from further comparison, ensuring an equitable comparison across successful distributions and sample sizes. The results are shown in Figure 12.

Computational time comparisons prove to be the most challenging to pin down, due to wide variations between distributions, sample sizes and random trials. However, Figure 12a demonstrates a clear advantage in the average computational time for $|Z_{4}|$, across all sample sizes. Once again, the number of outliers, which are compressed for clarity in Figure 12b, is roughly the same across the five scoring methods. However, $|Z_{AD}|$ has a higher range of typical runtimes, as well as higher averages in the smallest sample sizes. The KL-divergence comparisons shown in Figure 12c,d are less variable between scoring methods. A lower divergence between the estimate and the known reference distribution suggests a better estimate is being made. Figure 12c shows a decreasing KL-divergence with increasing sample size for all scoring methods, which demonstrates expected convergence, albeit with diminishing returns for larger sample sizes. Notably, $|Z_{AD}|$ produces slightly lower KL-divergence on average, compared to the other methods.

## 3. Discussion

Each of the five scoring methods have been evaluated when utilized within the PDFestimator and applied to the same distribution test set in terms of scalability, sensitivity, failure rate and KL-divergence. Each of the proposed measures have strengths and weaknesses in different areas. The $|Z_{AD}|$ measure produces the most accurate scaling and the lowest KL-divergence. The CS measure shows the greatest sensitivity for detecting small deviations from SURD. The $|Z_{LL}|$ method, although not a clear winner in any particular area, is notably well-performing in all tests. These results suggest a possible trade-off between a lower KL-divergence versus longer computational time with the $|Z_{AD}|$ scoring method. However, the slight benefit of a lower KL-divergence is arguably not worth the computational cost, particularly when also considering the higher failure rate. In contrast, the significantly low failure rate and fast performance times are strong arguments in favor of $|Z_{4}|$ as the preferred scoring method. However, this result is only true when the score of a sensitive measure is minimized, while the threshold to terminate is based on a less sensitive measure (see Section 4.7 in methods for details).

**Figure 13:**
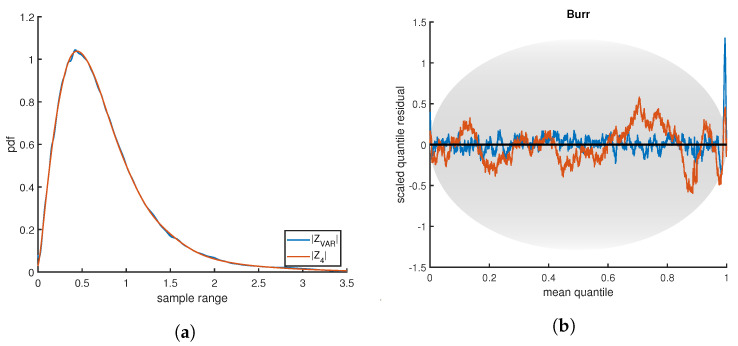
(**a**) Two density estimates are compared based on two different scoring functions. (**b**) The corresponding SQR-plots for each density estimate are shown. By eye, both density estimates look exceptionally good, but the SQR-plot has a strong peak representing error in the extreme tail of the distribution. The degree of error depends on the scoring function, but both scoring functions give qualitatively the same results.

Qualitative analysis is used to elucidate why $|Z_{4}|$ minimization is the best overall performer. The pdf and SQR for hundreds of different estimates were compared visually and robust trends were observed between the $|Z_{VAR}|$ and $|Z_{4}|$ methods. Figure 13a is a representative example, showing the density estimates for the Burr distribution at 100,000 samples. Although both estimates were terminated at the same quality level, the smooth curve found for $|Z_{4}|$ would be subjectively judged superior. However, there is nothing inherently or measurably incorrect about the small wiggles in the $|Z_{VAR}|$ estimate. Note that no smoothness conditions are enforced in the PDFestimator.

The SQR-plot, shown in Figure 13b, is especially insightful in evaluating the differences in this example. The Burr distribution is deceptively difficult to estimate accurately due to a heavy tail on the right. Both $|Z_{VAR}|$ and $|Z_{4}|$ fall mostly within the expected range, except for the sharp peak to the right corresponding to the long tail. Although the peak is more pronounced for $|Z_{VAR}|$, the more relevant point in this example is the shape of the entire SQR-plot. SQR for $|Z_{VAR}|$ contains scaled residuals close to zero, behavior virtually never observed in true SURD. Hence, this corresponds to over-fitting. This contrast in the SQR-plot between $|Z_{VAR}|$ and $|Z_{4}|$ is generally true with the following explanation.

The $|Z_{4}|$ scoring method uses the same threshold scoring as $|Z_{VAR}|$, but simultaneously seeks to minimize the variance from average, thus highly penalizing outliers to the expected z-score. The $|Z_{VAR}|$ method, by contrast, tends to over-fit some areas of the distribution of high density, attempting to compensate for areas of relatively low density where it deviates significantly. This often results in longer run times, many unnecessary Lagrange multipliers, less smooth estimates and unrealistic SQR-plots, as the NMEM algorithm attempts to improve inappropriately. For example, in the test shown in Figure 13, the number of Lagrange multipliers required for the $|Z_{VAR}|$ estimate was 141, whereas $|Z_{4}|$ required only 19. Therefore, it is easy to see why $|Z_{VAR}|$ took much longer to complete. This phenomenon is a general trend but it is exacerbated in cases where there are large sample sizes on distributions that have a combination of sharp peaks and heavy tails.

A surprising null result of this work is that the CS measure, custom designed to have the greatest overall sensitivity and selectivity, failed to be the best overall performer in practice when invoked in the PDFestimator. Although more investigation is required, all comparative results taken together suggest that the CS scoring function is the most sensitive but is over-designed for the capability of the random search optimization method currently employed in the PDFestimator. In the progression of improvements on pdf estimation, the results from the initial PDFestimator suggested that a more sensitive scoring function would improve performance. With that aim, more sensitive scoring functions have been determined and performance of the PDFestimator substantially improved. However, it appears the opposite is now true, requiring a shift in attention to optimize the optimizer, with access to a battery of available scoring functions. In preparation, another work (ZM, JF, DJ) optimizes the overall scheme by dividing the data into smaller blocks, which gives much greater speed and higher accuracy, while taking advantage of parallelization.

## 4. Methods

MATLAB 2019a (MathWorks, Natick, MA, USA) and the density estimation program “PDFestimator” were used to generate all the data presented in this work. The PDFestimator is a C++ program that JF and DJ developed as previously reported [11], which has the original Java program in supporting material. Upgrades on the PDFestimator are continuously being made on the BioMolecular Physics Group (BMPG) GitHub website, Available online:  https://github.com/BioMolecularPhysicsGroup-UNCC/PDF-Estimator, where the source code is freely available, including a MATLAB interface to the C++ program. An older C++ version is also available in R, https://cran.r-project.org/web/packages/PDFEstimator/index.html. The version on the public GitHub website is the most recent stable version that has been well tested.

### 4.1. Generating SURD and Scoring Function Evaluation

MATLAB was employed in numerical simulations to generate SURD. For a sample size *N*, the sort ordered sequence of numbers ${\left\{r_{k}\right\}}_{N}$ was used to evaluate each scoring function being considered. The same realization of SURD was assigned multiple scores to facilitate subsequent cross correlations.

### 4.2. Method for Partitioning Data

As previously explained in detail [11], sample sizes of N > 1025 were partitioned in the PDFestimator to achieve rapid calculations. The lowest and highest random number in the set ${\left\{r_{k}\right\}}_{N}$ define the boundaries of each partition. The random number closest to the median was also included. Partitions have an odd number of random numbers due to the recursive process of adding one additional random number between the previously selected random numbers in the current partition. Partition sizes follow the pattern of $3,5,9,17,33,\ldots 1+2^{n}$. A desired property of scoring functions is that they should maintain size invariance for all partitions. Scores for each measure were tracked for all partitions of size 1026 and greater, including the full data set, which is the last partition. For example, with N = 100,000 the scores for partitions of size Np = 1025, 2049, 4097, 8193, 16,385, 32,769, 65,537, 100,000 were calculated. Scores from different partitions were cross correlated in scatter plots.

### 4.3. Finite Size Corrections

For each partition of size $N_{p}$, including the last partition of size *N*, the scores were transformed to obtain data collapse. For all practical purposes finite size corrections were successfully achieved by shifting the average of a score to zero and normalizing the data by the standard deviation of the raw score. That is to say, the score, $S_{t}\left(N_{p}\right)$ for $N_{p}$ samples in the p-th partition, was a random variable. This score was transformed to a Z-value through the procedure $Z_{t}\left(N_{p}\right)=\left[S_{t}\left(N_{p}\right)-\mu_{t}\left(N_{p},N\right)\right]/\sigma_{t}\left(N_{p},N\right)$. Operationally, tens of thousands of random sequences of SURD were generated for each scoring function type to empirically estimate $\mu_{t}\left(N_{p},N\right)$ and $\sigma_{t}\left(N_{p},N\right)$. Note that $\mu_{t}\left(N_{p},N\right)$ and $\sigma_{t}\left(N_{p},N\right)$ were obtained using basic fitting tools in the MATLAB graphics interface, and these are reported in Table 1.

### 4.4. Decoy Generation

For each decoy the sort ordered sequence of numbers ${\left\{r_{k}^{o}\right\}}_{N}$ defining SURD was transformed into decoy-SURD, denoted as dSURD. This was accomplished by creating a model decoy cdf, $F_{d}\left(r\right)$. A new set of sort ordered random numbers was created by the 1 to 1 mapping ${\left\{r_{k}^{d}\right\}}_{N}=\;F_{d}\left({\left\{r_{k}^{o}\right\}}_{N}\right)$, yielding a dSURD realization per SURD realization. Different decoys were generated based on different types of perturbations, which must meet certain criteria. Let Δ(r) represent a perturbation to SURD, such that

(3) F(r)=r+Δ(r)

For the perturbation to be valid, the pdf given by $f_{d}\left(r\right)=\frac{dF_{d}\left(r\right)}{dr}$ must satisfy $f_{d}\left(r\right)\;\geq \;0$, which implies $1+\Delta^{\prime }\left(r\right)\geq 0$. The boundary conditions Δ(0)=Δ(1)=0 must also be imposed. With these conditions satisfied, decoys of a wide variety could be generated. Four types of decoys were created using this approach, listed in the first 4 rows of Table 3. In this approach, the amplitude of the perturbation is a parameter. A decoy that is marginally difficult to detect at sample size of $N_{d}$ has $\max\left(|\Delta |\right)=1/\sqrt{N_{d}}$. It will be challenging to discriminate between SURD and dSURD for $N<N_{d}$, and markedly distinguishable when $N/N_{d}\gg 1$.

Two additional types of decoys were also generated. First, $F_{d}\left(r\right)$ is set to a beta distribution cdf, denoted as $F_{\beta }\left(r|\alpha ,\beta \right)$. Therefore, the perturbation is given as $\Delta \left(r\right)=F_{\beta }\left(r|\alpha ,\beta \right)-r$. The α and β parameters were adjusted to tune detection difficulty, by systematically searching for pairs of α and β on a high resolution square grid to find when max(Δ) was at a level that was consistent with the targeted sample size, $N_{d}$. Second, a decoy can be defined by uniformly reducing fluctuations according to $r_{k}^{d}=r_{k}^{o}+p\left(r_{k}^{o}-\mu_{k}\right)$ where $\mu_{k}=k/\left(N+1\right)$. When p=0 the decoy was the same as SURD, but as p→1 the decoy retained no fluctuations. In this sense, this decoy type mimics extreme over-fitting, where *p* controls how much of the fluctuations are reduced.

### 4.5. ROC Curves

All ROC curves were generated according to the definition that the fraction of true positives (FTP) were plotted on the y-axis versus the fraction of false positives (FFP) plotted on the x-axis [22]. Note that alternative definitions for ROC are possible. To calculate FTP and FFP, a threshold score must be specified. If a score is below this threshold, the sort ordered sequence of numbers is predicted to be SURD. Conversely, if a score exceeds the threshold, the prediction is not SURD. As such, there are four possible outcomes. First, true SURD can be predicted as SURD or not, respectively, corresponding to a true positive (TP) or a false negative (FN). Second, dSURD can be predicted as SURD or not, respectively corresponding to a false positive (FP) or true negative (TN). All possible outcomes are tallied, such that FTP = TP/(TP + FN) and FFP = FP/(FP + TN). For a given threshold value, this calculation determines one point on the ROC curve. By considering a continuous range of possible thresholds, the entire ROC curve is constructed.

Procedurally, the data used to calculate the fractions of true and false positives that come from numerical simulations in MATLAB comprised 10,000 random SURD and dSURD pairs for sample sizes, N= 10, 50, 200, 1000, 5000, 20,000 and 100,000. About 60 different types of decoys were considered with diverse sets of parameters.

### 4.6. Distribution Test Set

To benchmark the effect of a scoring function on the performance of the PDFestimator, a diverse collection of distributions was selected and these are listed in Table 4. A MATLAB script was created to utilize built in functions dealing with statistical distributions to generate random samples of specified size. The random samples were subsequently processed by the PDFestimator to estimate the pdf, but for which the exact pdf is known. The set of possible distributions available for analysis cover a range of monomodal distributions that represent many types of features that include sharp peaks, heavy tails and multiple resolution scales. Some mixture models were also included that combine difficult distributions to create a greater challenge.

### 4.7. PDF Estimation Method

Each alternative scoring function, $\left\{|Z_{AD}|,|Z_{LL}|,|Z_{VAR}|,|Z_{4}|,CS\right\}$ was implemented in the PDFestimator and were evaluated separately. Factors confounding comparisons in performance include sample size, distribution type, selection of key factors to evaluate and consistency across multiple trials. To provide a quantitative synopsis of the strengths and weaknesses of the proposed scoring methods, large numbers of trials were conducted on the distribution test set listed in Table 4. The distribution test set increases atypical failures amongst the estimates because it is necessary to consider extreme scenarios to identify breaking points in each of the scoring methods. Nevertheless, easier distributions, such as Gaussian, uniform and exponential, were included. To wit, good performance of an estimator when applied to challenging cases should not suffer when applied to easier distributions.

As an inverse problem, density estimation applied to multiple random samples of the same size for any given distribution will generally produce variation amongst the estimates. For small samples, the pdf estimate must resist over-fitting, whereas large sample sizes create computational challenges that must trade between speed and accuracy. To monitor these issues, a large range of sample sizes were tested, each with 100 trials of an independently generated input sample data set. Specifically, 100 random samples were generated for each of the 25 distributions, for each of the following 11 sample sizes with N= 10, 50, 100, 500, 1000, 5000, 10,000, 50,000, 100,000, 500,000, 1,000,000. This produced a total of 27,500 test cases, each of which were estimated using five scoring methods. Statistics were collected and averaged over each of the 100 random sample sets.

Three key quantities were calculated for a quantitative comparison of the scoring methods—failure rate, computational time and Kullback-Leibler (KL) divergence [21]. It was found that the KL-divergence distance was not sensitive to the different scoring functions. Alternative information measures [23,24] could be considered in future work. Failure rate is expressed as a fraction of failures out of 100 random samples. The KL-divergence measures the difference between the estimate against the known reference distribution. Computational times and KL-divergences were averaged only for successful solutions and thus were not impacted by failures. A failure is automatically determined by the PDFestimator when a score does not reach a minimum threshold.

During an initial testing phase, it was found that the measures $Z_{t}$ and $|Z_{t}|$ for t= AD, LL, and VAR all worked successfully, which is not surprising considering the original measure, $Z_{LL}$, works markedly well. However, for the more sensitive measures, $Z_{4}$, $|Z_{4}|$ and CS, the PDFestimator failed consistently because the score rarely reached its target threshold, at least within a reasonable time. Therefore, a hybrid method was developed that minimizes a sensitive measure as usual, but the $|Z_{VAR}|$ measure was invoked to determine when to terminate. In tests of $|Z_{t}|$ for t= AD, LL or VAR, these measures were optimized and were simultaneously used as a stopping condition with a threshold of 0.66 corresponding to the 40% level in the cdf, which was the same level used previously [11]. All these measures have the same pdf and cdf, and thus the same threshold value. This threshold was used for $|Z_{VAR}|$ as a stopping condition when different scoring functions are minimized.

## 5. Conclusions

Several conclusions can be drawn from the large body of results presented. (1) The scaled quantile residual (SQR) is instrumental in assessing the quality of a pdf by means of visual inspection. The advantage of an SQR-plot over a traditional QQ-plot is that the displayed information is not only universal (distribution free), but importantly, sample size invariant; (2) It is possible to construct myriad scoring functions that are universal and sample size invariant based on quantitatively characterizing SQR. In particular, various measures can be developed based on mathematical properties of single order statistics (SOS) and/or double order statistics (DOS); (3) Finite size corrections can generally be applied to scoring functions so that their asymptotic properties can be utilized for finite size samples, as low as N=9; (4) Surprisingly, the scoring functions based on the Anderson-Daring test, quasi log-likelihood of SOS and the variance of SOS z-score —when applied to sampled uniform random data (SURD) share identical pdf for their scores for all practical purposes. Moreover, the scores are invariant across sample size and for different size partitions that sub-sample the input data; (5) The concept of decoy-SURD is introduced and a few methods are given for creating decoy-SURD (dSURD). The purpose of dSURD is to quantify the sensitivity and selectivity of a proposed scoring function using Receiver Operator Characteristics (ROC) or other means, such as machine learning. The usefulness of dSURD to quantify uncertainty in density estimation parallels the use of decoys in the field of protein structure prediction. That is, better scoring functions can be developed by focusing on how they discriminate between true SURD and dSURD; (6) Implementing a more sensitive scoring function in a method that estimates a pdf from random sampled data does not necessarily imply the process of estimation will be improved. There are many confounding factors that determine the ultimate performance characteristics of an algorithm for density estimation, since speed and accuracy need to be balanced for a practical software tool; (7) Minimizing either the $Z_{4}$ or $|Z_{4}|$ scores greatly improved the performance of the PDFestimator, a C++ program for univariate density estimation, compared to the initially used scoring function $Z_{LL}$.

In closing, a few research directions that can stem from this work are highlighted. Interestingly, the mean log ratio of nearest neighbor differences in sort ordered SURD, when taken from two disjoint subsets, is normally distributed (at least to a very good approximation). Unaware of an existing proof of this result, the empirical result suggests that a proof should be sought given that the literature contains many works that derive the pdf for ratios of random numbers that are distributed in a specific way. The results presented here can be applied to the problem of constructing a more sensitive distribution free “test for goodness of fit.” Essentially, this was the main objective that was addressed but here the emphasis was on how to better quantify uncertainty for the process of estimating a pdf for a random sample of data. Going forward, the universal sample size invariant measures developed here can be employed to test the similarity of two random samples of data.

## Figures and Tables

**Table 1:** Scoring function definitions and finite size corrections.

Anderson-Darling (AD) $S_{AD}=\frac{1}{N}\sum_{k=1}^{N}\left(1-2k\right)\left[\;log\left(r_{k}\right)+log\left(1-r_{n+1-k}\right)\right]\;$ $S_{AD}^{\prime }=S_{AD}-S_{AD}^{o}$ $\mu_{AD}^{\prime }=1-0.250/\sqrt{N}-0.667/N$ $\sigma_{AD}^{\prime }=0.761+0.025/\sqrt{N}$	Log-Likelihood (LL) $S_{LL}=\frac{1}{N}\sum_{k=1}^{N}log\left[\frac{N!}{(k-1)!(N-k)!}{{\left(r_{k}\right)}^{k-1}\left(1-r_{k}\right)}^{N-k}\right]$ $S_{LL}^{\prime }=S_{LL}-S_{LL}^{o}$ $\mu_{AD}^{\prime }=1-0.297/\sqrt{N}-5.180/N+7.56/N^{1.5}$ $\sigma_{AD}^{\prime }=0.761-0.120/\sqrt{N}-0.351/N$
mean variance (VAR) $S_{VAR}=\frac{1}{N}\sum_{k=1}^{N}z_{k}^{2}$ $\mu_{VAR}=1-0.003/\sqrt{N}$ $\sigma_{VAR}=0.757+0.312/\sqrt{N}+0.406/N$	generalized moment ($S_{0.5}$) $S_{0.5}=\;{\left(\frac{1}{N}\sum_{k=1}^{N}{\left|z_{k}\right|}^{0.5}\right)}^{2}$ $\mu_{0.5}=0.704-0.008/\sqrt{N}+0.009/N+0.52/N^{1.5}$ $\sigma_{0.5}=0.302+0.000/\sqrt{N}+0.313/N$
root mean square of log-ratio (RMSLR) $RMSLR={\left[\frac{1}{R}\sum_{\forall (i,j)}{\left(S_{LR}^{ij}\right)}^{2}\right]}^{\frac{1}{2}}$ where $R=N_{b}\left(N_{b}-1\right)/2$ for distinct pairs. $N_{b}$= number of blocks.	generalized moment ($S_{4}$) $S_{4}=\;{\left(\frac{1}{N}\sum_{k=1}^{N}{\left|z_{k}\right|}^{4}\right)}^{0.25}$ $\mu_{4}=\;1.153\;+\;0.129/\sqrt{N}\;-\;1.630/N\;+\;2.20/N^{1.5}$ $\sigma_{4}=\;0.345\;+\;0.303/\sqrt{N}\;+\;0.762/N\;\;-\;2.56/N^{1.5}$
mean log-ratio (SLR)	
$S_{LR}^{ij}=\;\frac{1}{m-1}\sum_{k=1}^{m-1}log\left(\frac{\delta_{k}^{i}}{\delta_{k}^{j}}\right)$ where m = block size for both the i-th and j-th blocks being compared.	
$\mu_{LR}=0$ Let $x=\left(N_{p}-1\right)/\left(N-1\right)$ to account for the number of subsamples within partition, p.	
$\sigma_{LR}=\sqrt{\frac{2}{m}}\left\{\begin{array}{cc} 1+0.1888x+1.754x^{2}-13.71x^{3}+44.49x^{4}-47.01x^{5}, & x<1/2\\ -4.952+29.12x-50.52x^{2}+38.95x^{4}-11.32x^{4}, & x\geq 1/2 \end{array}\right.$	

**Table 2:** Area under the ROC curves shown in Figure 10.

	N = 5000			N = 20,000			N = 100,000		
decoy	$|Z_{VAR}|$	$|Z_{4}|$	CS	$|Z_{VAR}|$	$|Z_{4}|$	CS	$|Z_{VAR}|$	$|Z_{4}|$	CS
dimple	0.50	0.50	0.55	0.49	0.49	0.61	0.46	0.46	0.96
pulse	0.49	0.49	0.55	0.48	0.48	0.65	0.43	0.49	0.99
wavelet	0.49	0.49	0.72	0.47	0.48	0.99	0.42	0.51	1.00
sine	0.47	0.46	1.00	0.42	0.55	1.00	0.94	0.99	1.00
beta	0.62	0.62	0.70	0.87	0.87	0.92	1.00	1.00	1.00
reduced	0.89	0.97	1.00	0.89	0.97	1.00	0.89	0.97	1.00

**Table 3:** Decoy type summary.

Decoy Name	Perturbation Equation	Parameters
dimple	$\Delta \left(r\right)=A\exp\left[\frac{-{\left(r-r_{o}\right)}^{2}}{2\sigma^{2}}\right]$	$A,r_{o},\sigma$
pulse	$\Delta \left(r\right)=A\left(\frac{r_{o}-r}{\sigma^{2}}\right)\exp\left[\frac{-{\left(r-r_{o}\right)}^{2}}{2\sigma^{2}}\right]$	$A,r_{o},\sigma$
wavelet	$\Delta \left(r\right)=A\sin\left(m\pi r\right)\exp\left[\frac{-{\left(r-r_{o}\right)}^{2}}{2\sigma^{2}}\right]$	$A,r_{o},\sigma ,m$
sine	$\Delta \left(r\right)=A\sin\left(m\pi r\right)$	*A*, *m*
beta distribution	$\Delta \left(r\right)=F_{\beta }\left(r|\alpha ,\beta \right)-r$	α,β
reduced fluctuations	$\Delta_{k}=p\left(r_{k}^{o}-\mu_{k}\right)$	*p*

**Table 4:** List of distribution types and corresponding parameters used to generate random data samples. Parameter and variable names correspond to the labeling scheme of MATLAB. For mixture distributions, subscripts indicate the distribution used to create the mixture with ordinal numbering, and under the *Scale Parameter* column, for mixture distributions $p_{i}$ is the mixing weight.

Distribution Name	Shape Parameter	Scale Parameter	Location Parameter
Beta	a=0.5	b=1.5	
Beta	a=2	b=0.5	
Beta	a=0.5	b=0.5	
Bimodal Normal	$\sigma_{1}=0.8$ $\sigma_{2}=0.3$	$p_{1}=0.65$ $p_{2}=0.35$	$\mu_{1}=2$ $\mu_{2}=6$
Birnbaum-Saunders	γ=0.5	β=1.5	
Birnbaum-Saunders and Stable	$\gamma_{1}=0.5$ $\alpha_{2}=0.5$ $\beta_{2}=0.5$	$\beta_{1}=1.5$ $\gamma_{2}=1$	$\delta_{2}=7$
Burr	c=2 k=2	α=1	
Exponential	μ=1		
Extreme-Value		σ=2	μ=1
Gamma	k=1	σ=2	μ=2
Generalized-Extreme-Value	a=2	b=2	b=2
Generalized-Pareto	k=2	σ=1	θ=0
Half Normal		σ=1	μ=0
Inverse Gaussian	λ=1	μ=5	
Normal	σ=1		μ=1
Normal Contaminated	$\sigma_{1}=2$ $\sigma_{2}=0.25$	$p_{1}=0.5$ $p_{2}=0.5$	$\mu_{1}=5$ $\mu_{2}=5$
Stable	α=0.5 β=0.05	γ=1	δ=4
Stable	α=0.2 β=0.05	γ=1	δ=4
Stable	$\alpha_{1}=0.5$ $\beta_{1}=0.05$ $\alpha_{2}=0.5$ $\beta_{2}=0.05$	$\gamma_{1}=1$ $\gamma_{2}=1$ $p_{1}=0.25$ $p_{2}=0.75$	$\delta_{1}=2$ $\delta_{2}=5$
Stable	$\alpha_{1}=0.5$ $\beta_{1}=0.05$ $\alpha_{2}=0.5$ $\beta_{2}=0.05$ $\beta_{3}=0.05$	$\gamma_{1}=1$ $\gamma_{2}=1$ $\gamma_{3}=1$ $p_{1}=0.25$ $p_{2}=0.5$ $p_{3}=0.25$	$\delta_{1}=2$ $\delta_{2}=5$ $\delta_{3}=8$
Trimodal Normal	$\sigma_{1}=0.5$ $\sigma_{2}=0.25$ $\sigma_{3}=0.5$	$p_{1}=0.\bar{3}$ $p_{2}=0.\bar{3}$ $p_{3}=0.\bar{3}$	$\mu_{1}=4$ $\mu_{2}=5$ $\mu_{3}=6$
t-Location Scale	ν=1	σ=0.5	μ=4
Uniform	l=4 u=8		
Uniform-Mix	$l_{1}=1$ $l_{2}=3.5$ $l_{3}=7$ $u_{1}=2$ $u_{2}=5.5$ $u_{3}=9$	$p_{1}=0.1\bar{6}$ $p_{2}=0.6\bar{6}$ $p_{3}=0.3\bar{6}$	
Uniform Periodic	$l_{1}=1$ $l_{2}=2.5$ $l_{3}=4$ $l_{4}=5.5$ $l_{5}=7$ $l_{6}=8.5$ $u_{1}=2$ $u_{2}=3.5$ $u_{3}=5$ $u_{4}=6.5$ $u_{5}=8$ $u_{6}=9.5$	$p_{1}=0.1\bar{6}$ $p_{2}=0.1\bar{6}$ $p_{3}=0.1\bar{6}$ $p_{4}=0.1\bar{6}$ $p_{5}=0.1\bar{6}$ $p_{6}=0.1\bar{6}$	
Weibull	b=2	a=1	
